# How helpful are Patient and Public Involvement strategic documents - Results of a framework analysis using 4Pi National Involvement Standards

**DOI:** 10.1186/s40900-019-0164-0

**Published:** 2019-11-04

**Authors:** Rachel Matthews, Meerat Kaur, Catherine French, Alison Baker, Julie Reed

**Affiliations:** 1 Interim Head UCL Centre for Co-Production in Health Research, UCL Culture, 38-50 Bidborough Street, London, WC1H 9BT UK; 2 NIHR CLAHRC Northwest London, Chelsea and Westminster Hospital, 369 Fulham Road, London, SW10 9NH UK; 3 Clinical Integration Lead, Guy’s and St Thomas’ NHS Foundation Trust, Education Centre, 75-79 York Road, London, SE1 7NJ UK

**Keywords:** Patient, Public, Involvement, Strategic, Framework method, Programme theory, Action effect method, Quality improvement

## Abstract

**Background:**

Patient and Public Involvement (PPI) strategic documents are viewed as an essential feature of organisational commitment to openness and transparency. They provide a mechanism to communicate opportunities for wider community influence in healthcare. The absence of documentation can be negatively interpreted, for example during regulatory inspection, as a lack of intent by organisations to collaborate with a broad constituency. Published literature paints a confusing picture of rationale and evidence that could provide the foundation for strategic action. This makes it difficult for those responsible for turning goals into meaningful involvement. We investigated how content is presented and organised in strategic documents. This pragmatic study is intended to stimulate reflective practice, promote debate and generate further inquiry with a wide audience.

**Methods:**

We created and iterated a framework adapted from 4Pi National Involvement Standards to analyse organisational PPI strategic documents against five domains which are principles, purpose, presence, process and impact. Fifteen strategic documents were grouped into four categories (acute care providers; clinical commissioning groups; community healthcare providers; and other) and included for analysis. A matrix was produced. By reading the matrix vertically (down) and horizontally (across), comparisons can be made between 4Pi domains and across organisations.

**Results:**

There was no discernible pattern between domains or between organisations. There was variation in the level to which criteria were met. No single strategy fully met the criteria for all five domains of 4Pi National Involvement Standards. The criteria for purpose was fully met in eight strategic documents. Only two documents fully met impact criteria. Four organisations showed better completeness with fully or partially met criteria across five domains. A single organisation partially met the criteria for all domains. The remaining 10 were unable to meet the criteria in at least one domain.

**Conclusion:**

Our findings align with published literature that suggests the underpinning rationale for PPI is confusing. A strategic aim is difficult to articulate. Context and complexity are at play making the sharing of generalisable knowledge elusive. We offer further critique about the value of these documents and consider: ‘is there an alternative approach to construct PPI strategy to generate theory, capture learning and evaluate effectiveness at the same time?’ We suggest testing the adoption of programme theory in PPI. The emergent nature and context sensitive features of programme theory enable curiosity, creativity and critical appraisal. It has the potential to release practitioners from the tokenistic cycle of monitoring and reporting and replace this with a richer understanding of ‘what’ works and ‘how’ tied to a ‘why’ – in order to achieve a shared aim that everyone can get behind.

## Plain English summary

The availability of organisational Patient and Public Involvement (PPI) strategic documents suggests that there is a commitment to work with a wider community to improve healthcare and strengthen health research. Drawing on our experience working with practitioners we noticed documents are not always available or written in a way a non-expert can understand. Proposed actions are not necessarily linked to a defined goal. This makes it difficult for those responsible for turning strategic goals into real action. People who could help, especially public contributors, may find it difficult to understand when, where and how their knowledge and experience could be best used. This led us to investigate further. We tested a framework adapted from 4Pi National Involvement Standards to methodically assess 15 documents from different healthcare organisations including those active in community care, research and education. We examined how document content is presented and organised. No single document met the full criteria for addressing the standards which are organised into five domains called principles, purpose, presence, process and impact. There was variation. We critique the value of these documents in practice and ask: ‘is there an alternative approach to construct PPI strategy to generate theory, capture learning and evaluate effectiveness at the same time?’ We open debate about alternative ways to show and evidence organisational practice and learning. We suggest an approach called ‘programme theory’ which has the potential to minimise tokenistic practice and connect learning about the ‘why’, ‘what’, and ‘how’ of PPI.

## Background

Patient and Public Involvement (PPI) strategic documents are viewed as an essential feature of organisational commitment to openness and transparency. They can provide a mechanism to communicate opportunities for wider community influence in healthcare. The presence of a PPI strategic document on a website or the production of this evidence on request is interpreted as a signal that efforts are being made to connect with people who use services or with those who may be able to contribute to the generation of new knowledge about health and healthcare, for example through research and quality improvement (QI) [1–3]. The absence of documentation can be negatively interpreted, for example during regulatory inspection, as a lack of intent by organisations to collaborate with a wider constituency [4]. The Oxford English Dictionary (OED) defines the word strategy as ‘a plan of action designed to achieve a long-term or overall aim’. We also note Mintzberg’s 5 P’s for strategy where he defines strategy as ‘some sort of consciously intended course of action’ and identifies features of plan, ploy, pattern, position and perspective [5]. Others have scrutinised the use of strategic documents in healthcare [6]. Whilst business and managerial definitions can be more expansive, we suggest that the OED definition is inclusive, jargon free and can be more readily understood by the general reader. It is this definition that informs our research. In this paper we favour the definition of involvement used by INVOLVE (the National Institute for Health Research [NIHR] centre that supports public involvement in the NHS, public health and social care research) which describes involvement as an activity that is done ‘with’ or ‘by’ patients and the public and not ‘to’ or ‘for’ or ‘about’ them.

Despite the proliferation of evidence, policy, guidance and toolkits about PPI in healthcare and research, published literature offers a complex and confusing picture about the underlying rationale for involvement practice [7–14]. Martin [8] provides a helpful discourse about the differing rationales for participation. Drawing on interdisciplinary literature, Martin explores the tensions between democratic and technocratic rationales. Accountability, legitimacy and representativeness are highlighted in relation to the democratic rationale while aspects of consumerism, quality improvement and increasing personalisation are linked to the technocratic perspective. Literature [15, 16] identifies different types of publics and draws attention to the perceived superiority of professional knowledge by comparison with experiential knowledge. Issues of structural and positional power are explored too. This makes the identification of an inclusive, easily understandable strategic aim for PPI in any organisational context elusive. Health reforms, increasing competitiveness in higher education institutions, and frequent restructuring contribute to uncertainty and confusion in defining strategic aims. For practitioners, it is challenging to identify robust actionable evidence, grounded in theory and adaptable to different contexts, that they can apply with confidence leading to more inclusive involvement approaches [17]. Similar challenges are faced in the field of QI where the perceived lack of rigour in reporting is thought to weaken its value and limit its adoption more widely across healthcare [18].

Against this complex background, we decided to further investigate documentary evidence to explore the content and construction of PPI strategic documents across a range of organisations. This was in part prompted by a challenge experienced in practice when invited to conduct an informal peer review of a draft strategy in a partner organisation. We noticed the proposed actions appeared disconnected from the overall aim. Documentary evidence is not a proxy for actual practice and it is not our intention to offer judgements about practice or the individuals responsible for the construction of documents. Our plan was two-fold. First to adapt and test an existing framework to inform analysis and second to methodically critique documents against this benchmark which attends to important features of meaningful involvement.

We identified the 4Pi National Involvement Standards (4Pi) [19] as a valuable guide in setting out the essential domains that could be present in a PPI strategic document. 4Pi provides a framework aimed at establishing good practice and for monitoring, assessing and evaluating involvement. The reasons for our selection in favour of 4Pi as opposed to other frameworks are:

- It possesses universal relevance;
- It is underpinned by experiential and research evidence;
- It is firmly grounded in service user experience and was constructed through partnership working;
- It explicitly states the core purpose of involvement to be improvement;
- The standards are written in plain English and offer information and explanations that are of benefit to people new to involvement;
- Mental health service user experience is at the forefront of championing active and progressive involvement in healthcare.

We were familiar with and had expertise in using 4Pi to support healthcare professionals and patients in QI teams frame their PPI plans, actions and impact assessments [20, 21]. 4Pi offered a way to analyse documentary evidence and to organise findings. We investigated how content is presented and organised in strategic documents and identified questions about the value of these documents in operational delivery. This pragmatic study is intended to stimulate reflective practice, promote debate and generate further inquiry with a wide audience.

## Methods

We selected the Framework Method which originated in social policy research [22]. This method is suitable for qualitative content analysis and supports the organisation of material into a matrix where rows represent cases and columns support codes. By using this method, we rapidly compared *vertically between* 4Pi domains and *horizontally across* each organisation. The 4Pi domains of principles, purpose, presence, process and impact were adapted as a coding framework. We developed an iterative and pragmatic search and sampling strategy based on available time and resource. This was conducted between July and September 2016. We aimed to source PPI strategic documents for 63 partner organisations associated with NIHR Collaboration for Leadership in Applied Health Research and Care Northwest London (CLAHRC NWL) (Table 1).

**Table 1:** Aims: National Institute for Health Research (NIHR) Collaboration for Leadership in Applied Health Research and Care (CLAHRC)

• Develop and conduct applied health research relevant across the NHS, and translate research findings into improved outcomes for patients • Create a distributed model for the conduct and application of applied health research that links those who conduct applied health research with all those who use it in practice across the health community • Create and embed approaches to research and its dissemination that are specifically designed to take account of the way that healthcare is delivered across the local Academic Health Science Network • Increase the country’s capacity to conduct high quality applied health research focused on the needs of patients, and particularly research targeted at chronic disease and public health interventions • Improve patient outcomes locally and across the wider NHS; and • contribute to the country’s growth by working with the life sciences industry	

Source: [23]

### Search

We used key search words ‘patient’, ‘public’, ‘engagement’, ‘involvement’, ‘strategy’, ‘experience’, ‘Northwest London’, and ‘healthcare’. We visited websites and used Google search engine to locate documents. Where documents were not found in accessible public domains, we identified personal contacts who worked with or were affiliated to the organisation, for example patient and public involvement leads or patients and carers. Professionals with similar remits to our own roles were approached too.

### Sample

Early search results excluded industry organisations (e.g. pharmaceutical, business consultancies, and media companies) as their broad remit and geographical reach extended beyond Northwest London. Voluntary, third sector and charitable organisations were excluded for similar reasons. Disruption caused by local authority restructuring and financial constraints at the time of the search made it difficult to find active websites with current strategic documents. Fifteen documents were selected for final analysis. Identifying features were removed to preserve organisational anonymity (Table 2). Documents were varied in title and content and some included operational and implementation features. None of them offered a definition of the term strategy.

**Table 2:** Source of documents included for final analysis

Source of documents included for final analysis		
Organisation	Group	n
Acute Care Providers (includes mental health and tertiary trusts)	A	4
Clinical Commissioning Groups (CCG)	B	6
Community Healthcare Providers	C	1
Other (includes ambulance, research and education)	D	4
Total		15

### Analysis

We created and iteratively tested an analysis framework adapted from the five domains of 4Pi (Table 3). In the early stages, an external NIHR colleague acted as an adviser, drawing on experience of reviewing similar documents. MK and RM analysed the same strategy (*n* = 1) against the criteria, discussing differences of opinion to inform any further adaptations. Following this test we introduced sub-questions to strengthen inter-rater reliability. MK and RM subsequently analysed three documents together: one acute trust; one clinical commissioning group; and one community healthcare trust (*n* = 3). No further iterations were made to the framework. Analysis was independently conducted for the remaining documents (*n* = 11). Further discussion was initiated with AB if there was perceived ambiguity and difference of opinion to reach consensus. NVivo 10 software was used to store, order and code data and to select relevant supporting quotes. A rating was assigned to each strategy and corresponding domain coding cell (Table 4). AB reviewed emerging results and final analysis offering her insight as a public contributor and carer. She has experience and knowledge as a public representative on partner organisation governance structures. This informed further sense-making to produce early drafts of the results and discussion.

**Table 3:** Adapted Analysis Framework using 4Pi National Involvement Standards [19]

Adapted Analysis Framework using 4Pi National Involvement Standards		
4Pi Domain	Definition used for analysis	Questions to support analysis
Principles	• A set of values that inform meaningful involvement	1. Are values identified? e.g. ‘equality and diversity impact assessments inform our strategy’ 2. Is there evidence that values influence the strategy? 3. Are principles stated?
Purpose	• Makes it explicit why people are involved • Describes why people are involved • Provides a rationale/goal for activity	1. Is there a purpose or aim? 2. Are objectives recorded?
Presence	• Describes which groups/people need to be involved to shape and achieve the stated purpose	1. Who is the strategy author? 2. Who has influenced the strategy? 3. Are target groups/populations identified? 4. Is information available about key contacts/partners?
Process	• Describes how involvement will happen • Sets out a series of relevant/appropriate methods or steps to achieve aim/objectives • Indicates opportunity for reflection/learning/evolution over time	1. Are plans described to achieve the purpose or aim? 2. Are there defined, time bound mechanisms to deliver the strategy? (Who, when, where, how) 3. Are reporting mechanisms in place to provide progress reports to all those involved? 4. Are accountability lines documented up to and including executive level?
Impact	• Describes the difference involvement will make (intended/short-medium-long-term)	1. Is there evidence of success/impact criteria? 2. Are there defined mechanisms to assess impact? 3. Are there defined mechanisms for measurement and/or evaluation?

**Table 4:** Rating assigned to each 4Pi National Involvement Standard Domain [19]

Rating assigned to each 4Pi National Involvement Standard Domain		
Rating	Code	Shared definition used by analysts
Unmet	U	• No evidence to address analysis questions
Partially met	P	• Ambiguous • Some evidence to address analysis questions • Insufficient explanation and detail
Fully met	F	• Clear • Enough evidence to address analysis questions • Sufficient explanation and detail

## Results

No single strategy fully met the criteria for all five domains of 4Pi (Table 5). The criteria for purpose was fully met by eight strategic documents (Table 5: A1; A4; B6; B7; B9; C11; D12; D13). Presence, process and impact, domains were weak; only two documents met the full criteria for impact (Table 5: B9; C11) and two documents met the full criteria for presence (Table 5: B9) and process (Table 5: D15), respectively. We present findings by domain and organisation.

**Table 5:** Rating assigned to PPI strategic documents by 4Pi domain and organisation

Rating by 4Pi domain and organisation					
Group/ID	Principles	Purpose	Presence	Process	Impact
A1	F	F	P	P	P
A2	F	P	U	U	P
A3	P	P	P	P	P
A4	U	F	P	P	U
B5	U	P	P	P	U
B6	U	F	U	P	U
B7	F	F	P	P	P
B8	U	U	P	P	U
B9	F	F	F	P	F
B10	U	U	P	P	U
C11	P	F	U	P	F
D12	U	F	P	P	U
D13	U	F	U	U	U
D14	U	P	P	U	P
D15	P	P	P	F	P

**Group A** Acute Care Providers (includes mental health and tertiary trusts)

**Group B** Clinical Commissioning Groups (CCG)

**Group C** Community Healthcare Providers

**Group D** Other (includes ambulance, research, and education)

Key: U, Unmet; P, Partially met; F, Fully met

### Domain 1: principles

There was no evidence from eight strategic documents that they addressed any aspect of principles or values to inform strategic direction. Of the four documents that fully articulated principles, three were influenced by or co-created with patients/public:

*“The Trust’s values state that, in everything we do, we will provide: Care – Helping people when they need us; treating people with compassion, dignity and respect; having pride in our work and our organisation.” [D15]*

*“Our journey to improve patient and staff experience and engagement began … with the launch of … values aimed at supporting a culture that puts patients first” [A1]*

### Domain 2: purpose

Thirteen strategic documents fully or partially met the criteria for purpose. For example, this aim is focused on improving patient experience:

*“The aim … is to understand the individual needs and fully support all patients and their carers at every point of care, staff and the public to work together … to lead the NHS in improving patient experience.” [A1]*

Other aims implied a technocratic purpose:

*“In implementing this strategy, the CCG will meet its statutory obligations for patient involvement and equality … (Health and Social Care Act 2012, s26)” [B8]*

*“We have a duty and obligation to inform, engage and consult with the public to ensure accountability and build trust.”[B10]*

PPI is also articulated as a mechanism through which change could be achieved to deliver more equitable care and health outcomes:

*“Bringing together the expertise of local people and professionals to champion and drive high quality health and wellbeing for all in our community and overcome health inequalities.” [B7]*

### Domain 3: presence

One strategic document fully met the criteria. Ten partially met the criteria and four did not meet it. There was some indication about who is involved but it was often vague and lacking in detail. There was little or no evidence to show any understanding of the demographics of local populations or service users that might inform the strategic document. If users were referred to, they were more likely to be presented as a homogenous group:

*“This strategy describes the shared vision of [organisation] and its User Panel” [B9]*

*“The User Panel has been central in defining the overall mission for our CCG” [B9]*

*“Co-production of our involvement strategy with the strategic lay forum – and … other patients, carers and local people … we have run two ‘co-design’ events to produce our longer-term involvement strategy and implementation plan.” [A3]*

The absence of specific target groups presents difficulties when selecting appropriate involvement mechanisms. There was limited assurance that involvement would be shaped through the knowledge and experience of their local population groups:

*“ … the PPE objective of ensuring inclusion of patients and public from across our constituent communities and groups and address health inequalities.” [B5]*

Some documents did name or acknowledge those who had contributed to the content but this was rare.

### Domain 4: process

One strategic document fully met the criteria, 11 partially met the criteria and three did not meet the criteria. There was variable evidence to explain how the overall purpose of the strategy was to be achieved. Whilst objectives and actions were included, it was difficult to understand how they linked to the overall aim and what the predicted impact of action might be. Reporting, monitoring and organisational accountability were variably explained. It was rare for documents to provide sufficient detail that would enable a reader with limited knowledge to understand what might happen in practice.

*“[organisation name] is committed to using PPE at all stages of the commissioning cycle and in particular: ... Specifying outcomes and procuring services: Engaging patients in service design and improvement...” [B10]*

*“As well as quarterly meetings with Theme leads and regular reports for the … Committee, we will hold workshops for PPI leads / champions within the [organisation], and use [Forum] to bring together stakeholders from across NW London.” [D12]*

### Domain 5: impact

Only two strategic documents fully met this criteria. It was hard to find examples of desired impact together with mechanisms that enabled it to be assessed, evaluated or measured.

Some strategic documents provided statements explaining ‘good’ implementation of the strategy [C11] and how it will make a difference [A1].

One strategy provided a useful framework to assess impact for those with knowledge and experience of involvement, and those who may need extra support. The strategy [C11] suggested how involvement would be evidenced at various organisational levels (e.g. “operational PPE [patient and public engagement]”; “corporate PPE”), alongside a traffic-light system for example:

*“Basic level: Starting off – Increased staff awareness of PPE requirements*

*Gold level: Exemplar – Service redesign activity conducted with evidenced input from service users” [C11]*

Whilst these may indicate the impact of improved processes, they do little to shed light on the desired outcome. Examples of evidence are stated as:

*“Minutes and documents must evidence that service users were involved, how they felt about their involvement and what impact it had” [A2]*

It was more common for documents to provide general statements about measuring impact but not enough clarity for anybody to meaningfully do so:

*“Measuring the level of impact people feel that they are achieving through our engagement activities” [B9]*

### Organisation type

There is no discernible pattern by organisation type. A1, B7, B9 and D15 show the best completeness with domains that are fully and partially completed and none that are unmet. A3 shows partial fulfilment across all five domains. All remaining organisations have domains which do not meet the criteria in at least one domain. Only C9 fully meets the presence criteria. D15 alone fully meets the process criteria. C8 and C11 fully meet the impact criteria.

## Discussion

Our results demonstrate a varied picture of involvement intent across different organisations. No single document addressed all five domains of 4Pi. Document readers are required to work hard to search for and disentangle the ‘why’ (strategic aim) and ‘how’ (plan of action) of involvement. Dense language has to be unpacked to gain an understanding of aspiration and direction. The documents differed in accessibility of language and content. Some were closer to Plain English and clearly explained NHS or organisational structures. Managerial and technical language with jargon and acronyms dominated the sample. Very few documents stated any meaningful detail about who was to be involved, for example by offering data about the local community or demographic information about the patient population across services. This was especially concerning when considering issues of inclusivity, equity and equality.

### Lack of clarity in rationale and presentation of content

In our introduction we identified that there is limited actionable evidence to support those tasked with developing, implementing and evaluating meaningful patient and public involvement. The identified policy tensions are reflected in the documents we investigated [8, 10, 12]. There are limited, absent, and confusing aims that are rarely, if ever, logically linked to the proposed process of involvement. There are unclear rationales for involvement and no attempts to reflect on the influence of structural, positional, and political power [11, 14, 24–26]. Recent findings about the availability of frameworks for use in PPI in the research context revealed 65 were available, although all of them had very little transferability beyond the groups that developed them [27]. Practitioners, researchers and public contributors who share responsibility for the operationalisation and delivery of strategic aims for PPI are faced with a complex challenge.

In drawing out the key results, the purpose domain of 4Pi is the one which was fully met in eight out 15 documents. This leaves seven documents without a clearly defined purpose. If we look at how impact is addressed, only two out of 15 documents fully met our criteria. Six documents were unable to meet the criteria for impact in any way. This raises questions about if and how purpose and impact can be logically linked. These domains are essential features of contemporary involvement practice. Each may be considered separately and can be valued differently. For example, the demonstration of impact continues to invite debate [28–32]. There are concerns about the emphasis on impact. Those who favour an emancipatory approach to involvement may be less willing to see this as the endpoint when participation in the process itself may be as or more valuable. Expressions of purpose may reflect aspects of both instrumental and emancipatory rationales. For example, some purpose statements in the sample used language that we interpreted as framed around the need to meet policy, legislative or regulatory incentives rather than an expression of future ambition underpinned by theory and articulated through a shared vision and values.

The relationship between the theory and practice of involvement has parallels with other fields, for example QI [33]. Whilst difficulties with reporting, impact and evaluation are documented [34–37], the generation of actionable theory to evidence why we should involve, and in what way, to achieve a desired outcome requires further exploration. If we pursue the comparison with QI, we note similar gaps between academic perspectives on the theory of involvement and the intuitive knowledge and experience of those who are engaged in its practice, especially those considered to have non-expert knowledge i.e. patients, carers and service users. The responsibility for the generation of theory is perceived to be in the academic domain. The role of patients and public contributors in generating insight about theory and practice is not well understood. Jones et al. [18] invited public contributor support when analysing qualitative data in a study to investigate the reporting of QI, although this group were excluded as potential interviewees. Barber et al. [38] report the nuanced and complementary roles patients and public contributors can play alongside professionals to achieve strategic goals, support practice and spread new ideas.

### Alternative to strategic documents

A gap between the theory and practice of involvement remains. This explains a commonly expressed frustration in the field when attempting to answer ‘why’ we should involve and difficulty in describing ‘how’ to do it with greater certainty that we will achieve meaningful results to improve care or strengthen research. We therefore open the debate by posing the question: ‘is there an alternative approach to construct PPI strategy to generate context specific theory, capture learning and evaluate effectiveness at the same time?’

We offer perspective from our applied health research programme where PPI is considered an integral component in supporting the translation of research evidence into practice using a QI approach [39]. We suggest the generation of programme theory. For example, the action effect method, or similar, could offer a way forward [40].

### Programme theory and the action effect method

There is growing interest in the relationship between PPI and QI. Bergerum and colleagues [3] report a literature review and realist synthesis to generate programme theory for active patient involvement in QI efforts. In our own programme we have resisted the construction of a ‘traditional’ strategy in favour of testing this approach. In QI practice the identification and articulation of programme theory can support effective initiatives if carried out with rigour [40, 41]. Programme theory is described as ‘including an agreed aim, anticipated cause/effect relationships between the interventions, and the aim and measures to monitor improvement’. In the context of programme theory, the action effect method and diagram specifically ‘provides a framework to guide the execution and evaluation of a QI initiative, a focal point for other QI methods and a communication tool to engage stakeholders’ [40]. If we repeat this description replacing PPI for QI, it offers a different prospect for those required to act who wish to deliberatively study its effect and for those who see an effect (desired or undesired) and need to understand the steps that produced it. The notion of communication can include the explicit opportunity to learn together, for example through small tests of change or Plan-Do-Study-Act (PDSA) cycles. If conducted with discipline and support, PDSAs offer a way to capture both predictable and unpredictable outcomes and events that are context specific and lead to valuable emergent learning [32, 42]. Small tests of change were used to successfully build a shared learning network hosted by NIHR CLAHRC NWL [43].

**Fig. 1:**
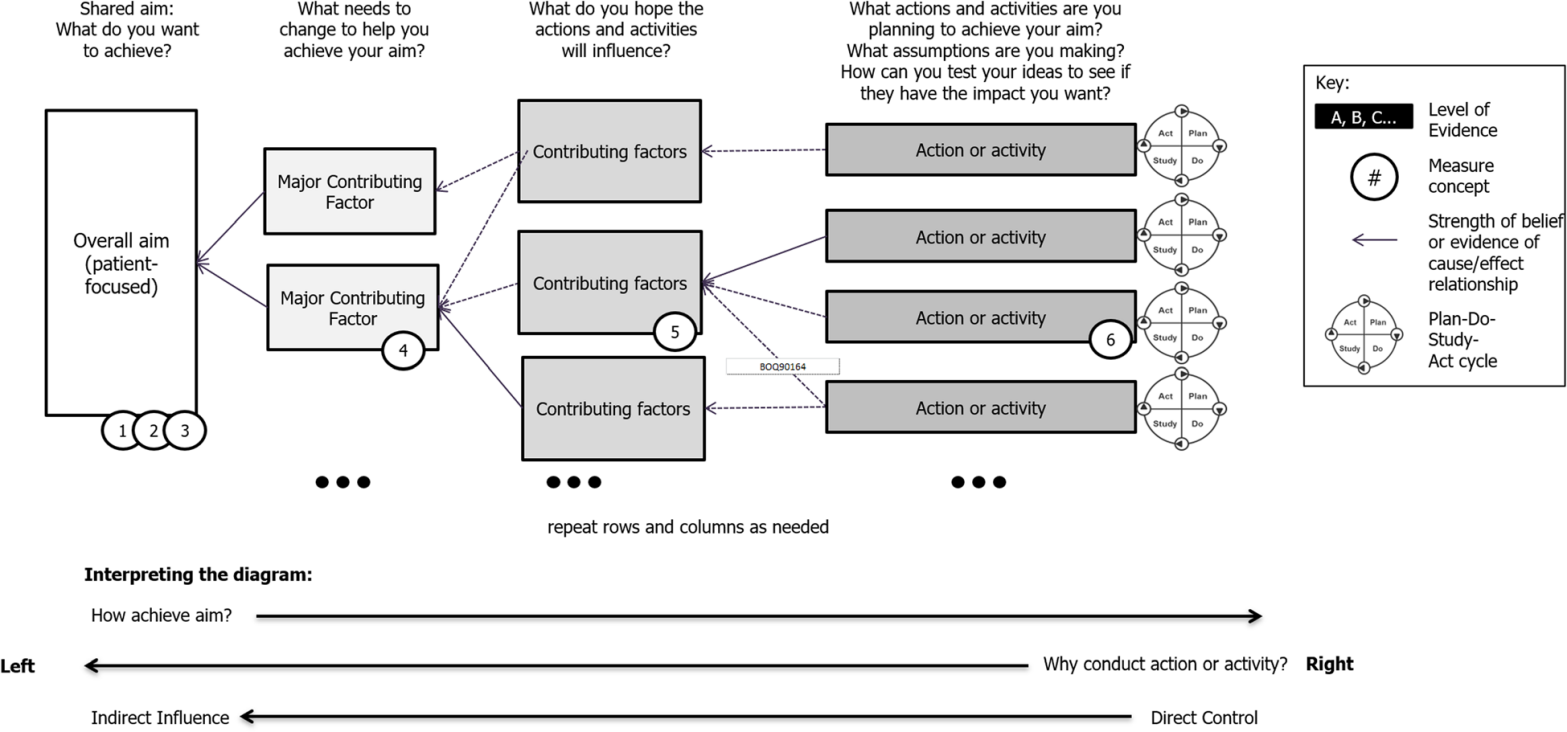
Schematic action effect diagram: guide to interpreting the components and overall structure of a typical action effect diagram [40]

The action effect diagram (Fig. 1) demonstrates, with horizontal arrows, the directions by which the ‘why’ and ‘how’ of an initiative works. While the notion of PPI as an intervention is contentious [32], we suggest that the discipline offered by articulating programme theory, for example through an action effect method, is worth considering. The action effect diagram explicitly states that all components are not within direct influence, indicating the roles of context and complexity. This frees practitioners to focus scarce resources where they are likely to have the most influence. The use of associated PDSA cycles has the potential to capture learning better. In our analysis we found very little evidence in the documents to explain ‘why’ PPI was being conducted and similarly scant identification of confident actions that might show others ‘how’. The action effect method or similar could ensure that these aspects are better identified and articulated and realistically tested rather than assumed to deliver results.

### Limitations of this study

There are limitations to our research. The study is localised, however the use of documentation in PPI is widespread and will be relevant internationally and for readers from different backgrounds. Further work could be conducted to test transferability to other settings. The final sample documents were not constructed with knowledge of or alignment with 4Pi. Our intention was to bring discipline and order to the subjective interpretation of qualitative information. We cannot rule out unconscious bias in our appraisal of the sample.

## Conclusion and recommendation

PPI is an established feature in healthcare policy and research in the UK and internationally. Patients, practitioners and researchers continue to grapple with the day to day reality of demonstrating meaningful practice that will further consolidate the value of involvement. In the absence of compelling actionable, context specific evidence, the gains are fragile. We open the debate about the limitations of PPI strategic documents and question the desirability of generating generalisable evidence and associated attempts to achieve consensus in how to capture impact. Testing the adoption of programme theory in the field of PPI, for example through the application of the action effect method, could introduce a structured approach to continually test and learn from practice. The emergent nature of programme theory enables shared curiosity, creativity and critical appraisal. It has the potential to release practitioners from the tokenistic cycle of monitoring and reporting strategic progress, replacing it with a richer understanding of ‘what’ works and ‘how’ tied to a ‘why’ – in order to achieve a shared aim that everyone can get behind.

## Data Availability

All reported data is in the manuscript. Relevant documents and qualitative data is held in Word documents, PDF files and NVivo version 10 files stored in password protected files on a shared drive at Imperial College London. Files are available on request from the corresponding author.

## Abbreviations

4Pi: 4Pi National Involvement Standards
CCG: Clinical Commissioning Group
CLAHRC: Collaboration for Leadership in Applied Health Research and Care
NIHR: National Institute for Health Research
NWL: Northwest London
OED: Oxford English Dictionary
PDSA: Plan, Do, Study, Act
PPE: Patient and Public Engagement
PPI: Patient and Public Involvement
QI: Quality Improvement
